# Improvement of urinary tract symptoms and quality of life in benign prostate hyperplasia patients associated with consumption of a newly developed whole tomato-based food supplement: a phase II prospective, randomized double-blinded, placebo-controlled study

**DOI:** 10.1186/s12967-020-02684-3

**Published:** 2021-01-06

**Authors:** Luigi Cormio, Beppe Calò, Ugo Falagario, Manuela Iezzi, Alessia Lamolinara, Paola Vitaglione, Giovanni Silecchia, Giuseppe Carrieri, Vincenzo Fogliano, Stefano Iacobelli, Pier Giorgio Natali, Mauro Piantelli

**Affiliations:** 1 Urology and Renal Transplantation Unit, Department of Medical and Surgical Sciences, University of Foggia, Foggia, Italy; 2 Bonomo Teaching Hospital, Andria (BAT), Italy; 3 Department of Medicine and Aging Sciences, Center for Advanced Studies and Technology (CAST), G.d’Annunzio University, Chieti, Italy; 4 Department of Agricultural Sciences, University of Naples, Portici, Italy; 5 Department of Agrotechnology and Food Science, Wageningen University, Wageningen, The Netherlands; 6 Janus Pharma S.r.l., Via Giacomo Peroni 386, 00131 Roma, Italy

**Keywords:** Benign prostate hyperplasia, Lower urinary tract symptoms, Tomato, Olive polyphenols, Food supplement

## Abstract

**Background:**

Benign prostatic hyperplasia (BPH) is the most common urologic disease among elderly men. The diagnosis of BPH is usually driven by lower urinary tract symptoms (LUTS) that can significantly affect patients’ quality of life. This phase II prospective, randomized double-blinded, placebo-controlled study aimed to determine the efficacy and safety of a novel whole tomato-based food supplement on LUTS of patients diagnosed with BPH.

**Methods:**

Forty consecutive patients with histologically proved BPH were randomized 1:1 to receive daily for 2 months a sachet (5 g) of a newly developed whole tomato food supplement (WTFS) (treatment = Group A) or placebo (Group B). Patients were asked to fill the International Prostatic Symptom Score (IPSS) questionnaire before and after treatment.

**Results:**

All but 1 patient in Group B successfully completed the scheduled regimen. No side effects were recorded. Unlike placebo, treatment significantly reduced (P < 0.0002) LUTS since mean IPSS decreased from 9.05 ± 1.15 to 7.15 ± 1.04 (paired t-test, two-tailed P-value < 0.001), and improved life quality (P < 0.0001). A trend toward a reduction of total PSA levels was observed in WTFS treated patients (8.98 ng/mL ± 1.52 vs 6.95 ± 0.76, P = 0.065), with changes being statistically significant only in the subgroup of patients with baseline levels above 10 ng/mL (18.5 ng/mL ± 2.7 vs 10.3 ± 2.1, P = 0.009).

**Conclusions:**

The new WTFS may represent a valid option for the treatment of symptomatic BPH patients. Unlike pharmacological treatments, the supplement is side effects free and highly accepted among patients.

## Introduction

Benign prostatic hyperplasia (BPH) affects aging men and is the most common urologic disease among elderly men [1]. BPH is the consequence of the proliferation of both epithelial and stromal cells from the transition zone and peri-urethral prostatic areas. It typically develops after the age of 40 years, ranging in prevalence from > 50% at 60 years to as high as 90% by 85 years [2].

The diagnosis of BPH is usually driven by lower urinary tract obstructive, and lower urinary tract symptoms (LUTS), i.e. urinary hesitancy, urgency, frequency, and post-void dribble. Pharmacological treatment possibilities include α-adrenergic antagonists or 5-α reductase inhibitors, however, one-third of patients with LUTS do not respond to either treatment approach [3] and a fraction of responders is penalized by the occurrence of side-effects. Patients who are resistant to medical treatment, or who become resistant to treatment over time will become candidates for surgical intervention to reduce LUTS severity.

Further understanding of the causes of LUTS will guide interventions to prevent LUTS or increase sensitivity to medical treatment. To date, there are multiple theories on the cellular and molecular processes underlying the pathogenesis of BPH leading to symptomatic disease. In addition to androgens, both chronic and acute inflammation can lead to events that can cause proliferation within prostatic tissue through a variety of mechanisms, notably oxidative stress [4, 5]. At present, non-steroidal anti-inflammatory drugs are used to improve urinary symptoms and flow measures, but their long-term effectiveness and safety are not known [6].

BPH is also associated with obesity and related pathologies. However, the biological pathways linking obesity and BPH are poorly understood. The centralized adipose deposition was associated with the severity of prostate tissue inflammation and LUTS and an approach to minimize centralized fat deposition may reduce LUTS severity in BPH patients [7]. Phytocompounds in the form of plant portions or extracts are widely used in the treatment of prostate diseases [8]. In this context, a large number of anti-inflammatory compounds have been identified in tomato extracts [9, 10]; moreover, tomato consumption reduces inflammation by decreasing inflammatory cytokines in overweight and obese men [11, 12].

Along this line of investigation, we have observed that a diet enriched with a 10% whole tomato [13] vs a tomato-free control diet increased the anti-oxidant serum activity and reduced serum levels of inflammatory biomarkers in the transgenic adenocarcinoma mouse prostate model (TRAMP) of prostate carcinogenesis, even before the appearance of cancer [14]. As a result, a decrease in cancer incidence and mortality was observed. In this regard, the role of lycopene is relevant, as a lycopene-poor tomato supplement failed to duplicate these findings [15]. However, the antineoplastic activity of the whole tomato preparations cannot be explained by the lycopene content alone [16–18], and constituents already present in small amounts or newly formed during processing also contribute to their final in vivo efficacy [19].

Recently, a whole tomato-based food supplement (WTBS) has been developed and registered by the Italian Health Ministry (registration n. 68843, 2018–19) with the claims “antioxidant” and”prostate health”. In a pilot study, we demonstrated that new WTFS, at the dosage of one sachet/day for 2 months, decreased LUTS in about 80% of BPH patients [20]. The present phase II prospective, randomized, double-blinded, placebo-controlled study aimed to determine the efficacy and safety of the novel whole tomato-based food supplement in reducing LUTS of patients with histologically proven BPH.

### Materials, methods, and patients

The WTFS which is produced by a patented solvent-free process resulting in increased bioavailability of antioxidant and anti-inflammatory tomato micronutrients: carotenoids, main lycopene with increased cis-configuration, flavonoids, and ketosamines is added with a small percentage of olive polyphenols [21]. The composition of the WTFS is reported in Tables 1 and 2, respectively.

**Table 1:** Tomato powder composition (for 100 g)

Carbohydrates (g)	64.5
Proteins (g)	10.2
Lipids (g)	3.4
Carotenoids (mg)	500
Lycopene isomers (mg)	190
Alpha-tocopherol (mg)	2.3
Total flavonoids (mg)	200
Ketosamines (mg)	8
Humidity (g)	< 5

**Table 2:** Polyphenolic content in olive extract (w/w)

Oleuropeinaglycon	6%
Ligstrosideaglycon	2%
Oleuropeindialdialdehydeaglycon	6%
Ligstrosidedialdehydeaglycon	7%
Verbascoside	6%
Pinoresinol and Deacetoxypinoresinol	5%
Tyrosol	3%
Hydroxytyrosol	10%
Unidentified polyphenols	8%

### Patients and methods

Forty consecutive patients having undergone prostate biopsy (PBX) at our Institution and having been diagnosed with BPH were included in a phase II prospective, randomized double-blinded, placebo-controlled study. The study protocol was approved by the University of Foggia Ethics Committee (Nov. 8, 2017; Registration Number 871-16) and was carried out in agreement with the provisions of the Declaration of Helsinki. Written informed consent was obtained from all patients.

Indications for trans-rectal ultrasound-guided PBX were increased serum prostate specific antigen (PSA) (≥ 4 ng/mL) and/or abnormal digital rectal examination. The inflammation present in prostatic biopsies was assessed and graded according to Irani’s score for both the histologic inflammation grading (extension of inflammatory cells, range 0–3) and aggressiveness (the effect of inflammatory cells on prostate tissue, range 0–3) [22].

Patients were randomized 1:1 into two Groups: Group A received one oral sachet of WTFS (5 g) every 24 h dissolved in water with no relation with meals, for 2 months while Group B received the placebo. Placebo consisted of orange/maltodextrin. The lycopene content in the WTSF sachet consisted of 9.5 mg isomers and of 12.5 all-trans, 1.75 5-cis, contained in the carotenoid fraction of the product. Patients reporting a history of hypersensitivity to tomato, inflammatory diseases of the urogenital tract (i.e. orchitis, epididymitis, or both), and malabsorption syndrome were excluded.

Patients had to fill the International Prostate Symptom Score (IPSS) questionnaire before and after treatment. Moreover, patients’ sera were collected before and after treatment, and 0.5 mL aliquots were immediately frozen and stored at − 80 °C until processing**.** Quantitation of total and free PSA as well as of cytokines/growth factors (IL-1, IL-6, IL-8, IL-17, IL-18, Angiopoietin 2, VEGF A) were performed by a multiplex assay (Milliplex®, Merck Life Science, Milano, Italy).

**Fig. 1:**
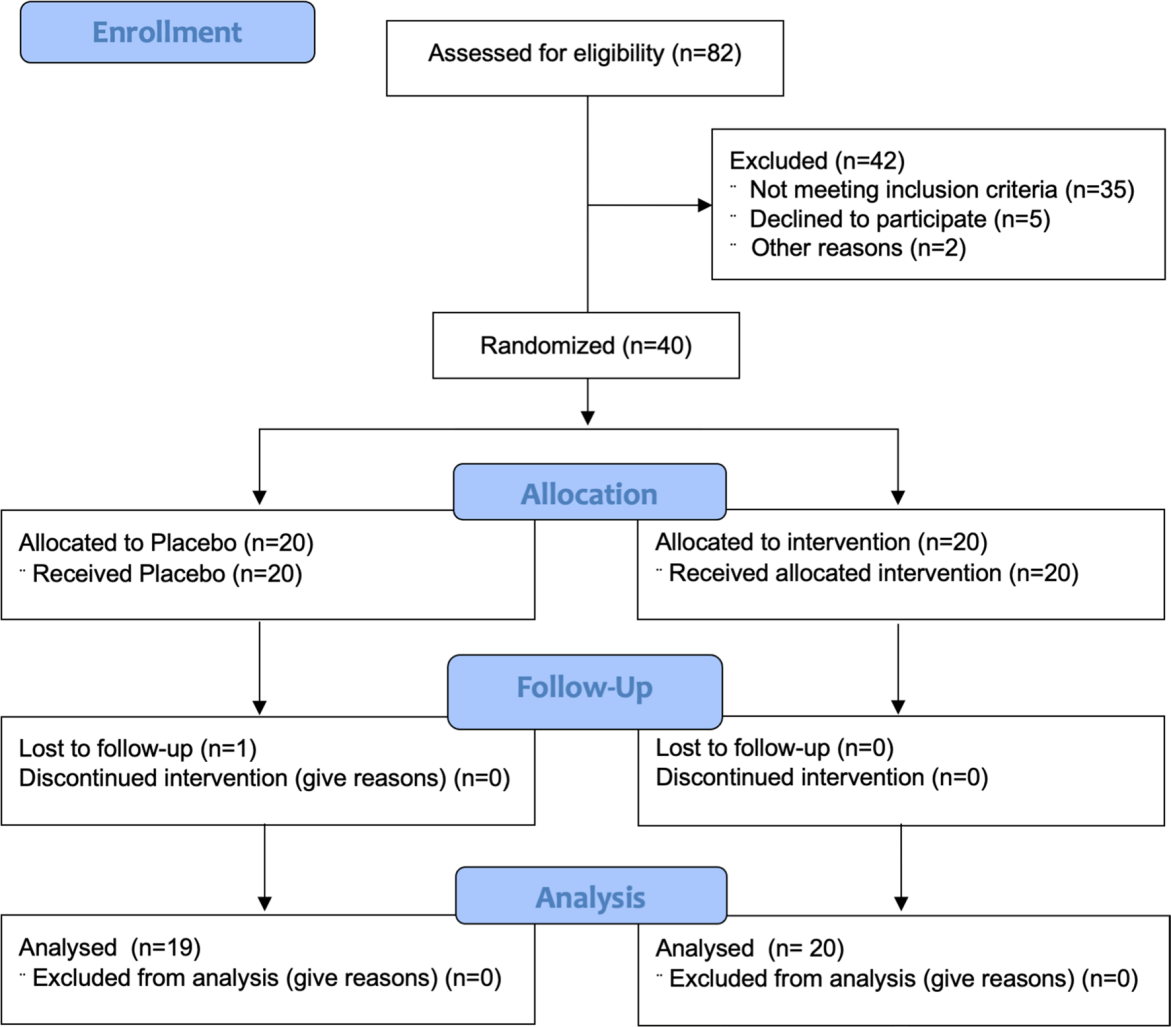
CONSORT 2010 Flow Diagram

The sample size was calculated assuming a 20% reduction in IPSS and an 80% study power; based on this data, 15 patients per group were needed. Therefore, 20 patients per Group were planned. A study flow diagram in agreement with the CONSORT guidelines is shown in Fig. 1.

The primary study endpoint was testing changes in LUTS as assessed by the IPSS questionnaire and PSA [23]. IPSS is based on the answer to seven questions concerning urinary symptoms and is the only questionnaire validated by WHO in the Italian language. Each question is assigned points from 0 to 5, indicating the increasing severity of a particular symptom. The total score, therefore, ranges from 0 to 35 (from asymptomatic to very symptomatic) and patients can be classified as follows: 0–7 = mildly symptomatic; 8–19 = moderately symptomatic; 20–35 = severely symptomatic.

## Results

There was no difference in terms of age, prostate volume, prostate inflammation, and serum PSA levels (Table 3) between treated (Group A) and control patients (Group B).

**Table 3:** Patients baseline characteristics

	Group A	Group B	P-value
Age	*65.6 ± 5.1	64.1 ± 8.2	0.468
Prostate volume (mL)	57.3 ± 7.1	59.8 ± 6.5	0.244
Prostate phlogosis (IRANI score)	2.5 ± 1.3	2.0 ± 1.2	0.182
Total PSA (ng/mL)	8.8 ± 1.5	6.6 ± 1.1	0.229

^*^All values are expressed as mean ± SE

One patient in Group B was lost at follow-up; all the others completed the study reporting no side effects. Patients in Group A experienced a significant reduction in mean IPSS 9.05 ± 1.15 (range 16–2) and 7.15 ± 1.04 (range 14–2), respectively (paired t-test, two-tailed P < 0.001) whereas patients in Group B did not (13.89 ± 6.45 vs 13.79 ± 6.29, P = 0.607)*.* The effects of treatment on the symptoms contributing to the IPSS score are detailed in Table 4. In particular, the WTFS strongly reduced both urination frequency (P = 0.002) and urgency (P < 0.0001). As a consequence, a significant improvement in life quality was referred by WTFS-assuming patients (P < 0.0001; Table 4). Quality of life was not affected by placebo (2.32 ± 0.29 vs 2.42 ± 0.26, P = 0.330).

**Table 4:** Effects of WTFS treatment on IPSS symptoms

Symptoms:	Pre-treatment score	Post-treatment score	P-value
1 Incomplete emptying	1.58 ± 0.25*	1.63 ± 0.23	P = 0.577
2 Frequency	1.40 ± 0.22	0.45 ± 0.18	*P* = *0.002*
3 Intermittency	1.55 ± 0.22	1.65 ± 0.25	P = 0.428
4 Urgency	1.16 ± 0.21	0.42 ± 0.19	*P* < *0.0001*
5 Weak Stream	1.50 ± 0.22	1.70 ± 0.24	P = 0.162
6 Straining	1.15 ± 0.22	1.05 ± 0.22	P = 0.163
7 Nicturia	0.80 ± 0.20	0.45 ± 0.18	*P* = *0.005*
Quality of life	2.45 ± 0.34	1.50 ± 0.27	*P* < *0.0001*

^*^All values are expressed as mean ± SE

**Fig. 2:**
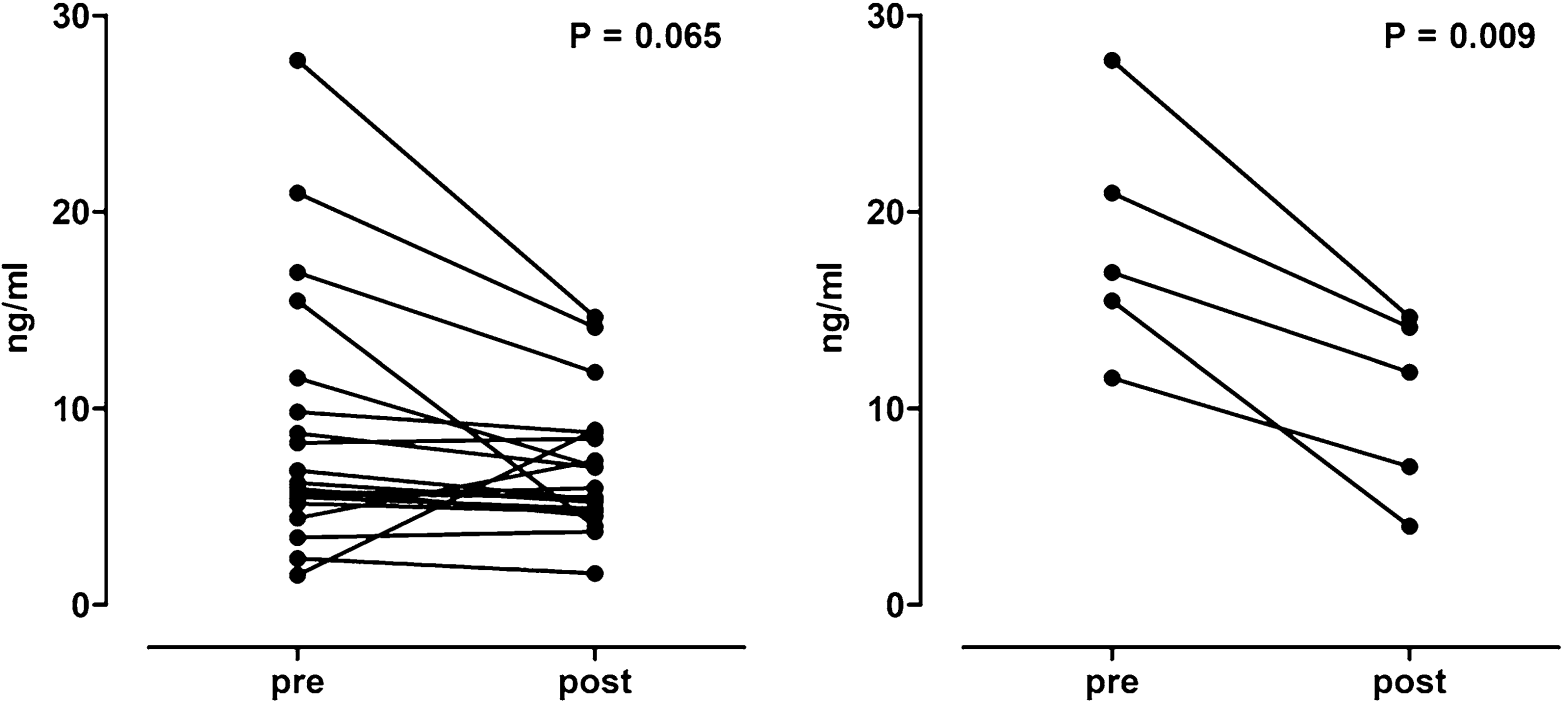
Total PSA levels pre and WTFS treatment. All patients (left panel) and patients with PSA > 10 ng/mL (right panel)

A trend toward a reduction of total PSA levels was observed in WTFS treated subjects (8.98 ng/mL ± 1.52 vs 6.95 ± 0.75, P = 0.065) (Fig. 2, left). Actually, this trend was sustained by the significant reduction of PSA concentrations seen in the five patients, (2 obese, 2 over- and 1 normal-weight), with basal levels > 10 ng/mL (18.520 ng/mL ± 2.747 vs 10.323 ng/mL ± 2.073, P = 0.009) (Fig. 2, right). On the other hand, at the end of the observation period, a trend toward an increase of total PSA was seen in placebo-treated subject (6.83 ± 1.09 vs 8.05 ± 0.96, P = 0.114).

Free PSA concentrations were not modified by the treatment (1.35 ± 0.24 vs 1.42 ± 0.198, P = 0.70). In addition, free/total PSA ratios were not significantly different before and after the WTFS treatment (17.11 ± 1.89 vs 22.38 ± 2.70, P = 0.117), and this is also true in the case of overweight/obese patients with basal levels of total PSA > 10 ng/mL (14.65 ± 3.39 vs 23.20 ± 4.63, P = 0.175).

## Discussion

The newly developed WTFS is a mixture of whole tomato powder and a polyphenolic extract from olives. The original tomato powder was firstly used to produce food for special medical purposes (FSMP) and investigated as adjuvant therapy in subjects affected by chronic hepatitis C. This FSMP was effective in preventing carotenoid serum depletion and improving the oxidative status during antiviral therapy [13]. Then, the anti-tumor activity of the tomato powder was evaluated in a transgenic mouse model of prostate carcinogenesis (TRAMP) [14]. In this model, feeding mice with the tomato powder significantly delayed tumor progression and decreased both the incidence of poorly differentiated carcinoma and mortality. Additionally, in agreement with several other reports, tomato supplementation was found to reduce the levels of circulating inflammatory/angiogenic cytokines as VEGF, TNF alpha, and IL-6 in several experimental models [24–26]. The patented method to produce the WTFS leads to a final product enriched in bioactive compounds, i.e. olive’s polyphenols which have been found more active than the tomato powder alone in reducing serum levels of IL-6 and VEGF in TRAMP mice [21]. Serum measurement of a panel of inflammatory cytokines in the pretreatment sera did not show any significant derangement (data not shown). This does not come unexpectedly since the prostate inflammation in BPH patients is unlikely to be mirrored in circulation.

Chronic prostatitis/chronic pelvic pain syndrome (CP/CPPS) is a urinary disorder that afflicts patients due to various discomforts. It is pressing and meaningful to develop novel and effective treatments because of the uncertain etiology and dismal therapeutic effect of CP/CPPS. In the absence of a standard treatment [27], the use of substances like antioxidants that may stop or potentially reverse the deleterious effects of inflammation is particularly attractive. Tomatoes and olives are the sources of complexes of micronutrients endowed with well-known strong antioxidants and anti-inflammatory activity, with properties potentially useful in protecting DNA and other cell constituents from oxidation [28, 29].

Here we demonstrate that the new WTFS determines a significant improvement of urinary tract symptoms and quality of life in BPH patients, thus representing a suitable alternative option to the pharmacological treatment of this age-related pathology. Although the molecular mechanisms underlying this clinical benefit remains to be investigated, in a rat model lycopene was found to attenuate chronic prostatitis/chronic pelvic pain syndrome by inhibiting oxidative stress and inflammation via the interaction of NF-κB, MAPKs, and Nrf2 signaling pathways [30].

Indeed, in addition to lycopene, tomatoes contain within their matrix other bioactive compounds that are responsible for the health effects associated with the constant consumption of the fruit, which cannot be accounted for solely by lycopene [18, 31]. The patented WTFS production method, not only generates more bioavailable cis-lycopene and increased tomato phenolic components but produces frus-his compounds that further increase tomato health-preserving properties [19]. Thus this WTFS is likely to possess a spectrum of cooperative mechanisms. In view that the increase of the epithelial and stromal components in BPH is not paralleled by noticeable proliferation, the hyperplastic lesions have been interpreted as the result of impaired programmed cell death mechanism [32, 33]. In this regard, tomato sauce has been reported to increased apoptosis in different experimental models [34, 35]. Stromal cells are responsible for the androgen-mediated glandular epithelium growth. Although a defined molecular pathway has not been yet defined, tomato and lycopene have been reported to down-regulate androgen metabolism and signaling [36].

Inflammation is a common histopathological finding in BPH, in absence of prostatic cancer or clinical prostatitis. It is a condition of which subclinical inflammation may be associated with a rise in serum PSA levels [37]. However weak is the correlation between PSA levels and the active or chronic inflammation and no significant correlation exists between the active or chronic histopathological inflammation and IPSS [38]. Also, in this regard nutritional interventions with tomato-products reduced PSA levels in subgroups of prostate patients, while lycopene supplements or extracts were found to be less effective [39–42].

Also, the mix of polyphenols from tomatoes and olive vegetation water can certainly contribute to the anti-inflammatory properties of the WTFS [43, 44].

This new food supplement can modulate the total PSA concentrations even in patients with BPH. We have observed an increasing total PSA trend in the placebo group (P = 0.080), probably as the consequence of the biopsy procedure. Indeed, cystoscopy can increase serum PSA levels fourfold, while needle biopsies and transurethral resection can temporarily increase PSA levels up to 50-fold, all as a result of increased PSA leakage into the serum. Besides, the relatively long half-life of PSA may lead to a consistent delay before serum PSA returns to a baseline level after transurethral prostatectomy, or infection [45]. On the contrary, a trend toward a reduction of total PSA levels was observed in WTFS treated patients (P = 0.096), which was sustained by the significant reduction of total PSA concentrations seen in the patients with high basal levels (> 10 ng/mL; P = 0.009).

Although BPH and prostate cancer (PCa) share features such as hormone-dependent growth and response to treatment with anti-androgen therapy, BPH is not considered a premalignant lesion. However, in a nationwide cohort study involving over 3 million men followed for up to 27 years, clinical BPH was associated with a two- to three-fold increased risk of PCa incidence and with a two- to eight-fold increased risk of PCa mortality [46]. Because PSA determinations when properly utilized may contribute to the diagnosis and follow-up of prostate cancer [47], the effect of tomato treatment on total PSA serum levels could interfere with clinical management. However, the determination of the percentage of free PSA and free/total PSA ratio appears to be a helpful method for enhancing the specificity of total PSA evaluation [48, 49] and WTFS does not change free PSA and free/total PSA values.

The overall tomato consumption may bear harmful effects on human health such as gastroesophageal reflux disease, irritable bowel syndrome, kidney stones, and some urinary problems [50]. In this context, it is worthy to be noted that, due to the preparation procedures, this WTFS does not contain organic acids (citric and malic acids), significant potassium/oxalate concentration and tomato skin/seeds, responsible for these side effects.

Based on recent human experimentation [51] this novel dietary supplement, which is phytosterols-free may help to maintain prostate health and can contribute to the beneficial effect of adhering to the WCRF/AICR recommendations [52], by itself, when complemented with current treatments [53] and by adopting healthy lifestyles. Furthermore, because of its high deliverable antioxidant activity, the consumption of the WTFS can be advantageous in contrasting the unhealthy effects of the excess production of free radicals induced by a variety of risk factors, including the metabolic syndrome often associated with BPH [25, 54].

Although tomatoes provide most of the dietary antioxidants in the Mediterranean-style diet, their culinary use is often associated with the consumption of carbohydrates rich meals (e.g. pasta, pizza), and high calories uptake (olive oil), thus representing a risk factor for individuals with excessive body mass, obesity, and metabolic syndrome. In this regard, the WTFS is an exemplary source of a complex of antioxidants. Furthermore, this food supplement may represent an ideal candidate to develop a variety of tomato-based enriched foods to delay the onset and/or to attenuate the course of age-related chronic degenerative disease [55, 56].

## Conclusions

The newly developed WTFS may represent an efficient option for the treatment of symptomatic BPH patients. Unlike pharmacological treatments, this supplement is side effects free and highly accepted among patients.

## Data Availability

Not applicable.

## Abbreviations

WTFS: Whole tomato food supplement
BPH: Benign prostate hyperplasia
LUTS: Lower urinary tract symptoms
PSA: Prostate-specific antigen
IPSS: International Prostatic Symptoms Score
